# Ovarian hyperstimulation closely associated with resumption of follicular growth after chemotherapy during tamoxifen treatment in premenopausal women with breast cancer: a multicenter retrospective cohort study

**DOI:** 10.1186/s12885-020-6549-5

**Published:** 2020-01-29

**Authors:** Rena Yamazaki, Masafumi Inokuchi, Satoko Ishikawa, Takuya Ayabe, Hiromitsu Jinno, Takashi Iizuka, Masanori Ono, Subaru Myojo, Soko Uchida, Toshiya Matsuzaki, Akira Tangoku, Masato Kita, Tomoharu Sugie, Hiroshi Fujiwara

**Affiliations:** 1 Department of Obstetrics and Gynecology, Kanazawa University Graduate School of Medical Science, 13-1 Takaramachi, Kanazawa, Ishikawa 920-8641 Japan; 2 Department of Breast and Endocrine Surgery, Kanazawa Medical University, Kanazawa, Japan; 3 Department of Breast Oncology, Division of Cancer Medicine, Kanazawa University Graduate School of Medical Science, Kanazawa, Japan; 4 Department of Obstetrics and Gynecology, Teikyo University School of Medicine, Tokyo, Japan; 5 Department of Surgery, Teikyo University School of Medicine, Tokyo, Japan; 6 Department of Gynecology, National Hospital Organization Fukuokahigashi Medical Center, Koga, Japan; 7 Department of Obstetrics and Gynecology, Graduate School of Biomedical Sciences, Tokushima University, Takushima, Japan; 8 Department of Thoracic, Endocrine Surgery and Oncology, Graduate School of Biomedical Sciences, Tokushima University, Takushima, Japan; 9 Department of Obstetrics and Gynecology, Kansai Medical University, Hirakata, Japan; 10 Department of Breast Surgery, Kansai Medical University Hospital, Hirakata, Japan

**Keywords:** Breast cancer, Chemotherapy, Estradiol, Gonadotropin-releasing hormone, Ovarian hyperstimulation, Tamoxifen

## Abstract

**Background:**

We previously reported that tamoxifen (TAM)-induced ovarian hyperstimulation (OHS) is associated with high serum concentrations of estradiol in premenopausal women with breast cancer. To investigate risk factors for TAM-induced OHS, we performed a retrospective multicenter study.

**Methods:**

Premenopausal patients who received surgical therapy for endocrine-dependent breast cancer (*n* = 235) were recruited in this study and classified into 4 groups: group A, treated with TAM alone; group B, TAM treatment after 2-year-combined therapy with a gonadotropin-releasing hormone (Gn-RH) agonist; group C, TAM treatment after chemotherapy; group D, 5-year-combined therapy with TAM and a Gn-RH agonist. A serum estradiol value of more than 300 pg/mL or mean follicular diameter of more than 30 mm was defined as OHS.

**Results:**

The incidence of OHS in group A (*n* = 13/26, 50.0%) was significantly higher than those in group B (*n* = 17/63, 27.0%), group C (*n* = 20/110, 18.2%), and group D (*n* = 0/36, 0%). The incidence of OHS was significantly correlated with aging, and the median serum concentration of estradiol in the presence of OHS was 823.0 pg/mL. The incidence of OHS (less than 47 years old) was 62.5% in group A, 48.6% in group B, and 28.2% in group C, respectively. Notably, the incidence rate of OHS following amenorrhea in group C (*n* = 13/20, 65.0%) was significantly higher than that in group B (*n* = 1/17, 5.9%).

**Conclusions:**

These findings indicate that the onset of OHS following amenorrhea was common in the post-chemotherapeutic group, while its ratio was low in the group after Gn-RH analog treatment, suggesting that combined treatment-based management involving TAM therapy is necessary for premenopausal patients with breast cancer.

## Background

Over the last few decades, the incidence rate of breast cancer has grown markedly in Japan, with a concomitant increase in the premenopausal occurrence of estrogen-dependent breast cancer [1, 2]. Tamoxifen (TAM) is a nonsteroidal anti-estrogen drug [3] and its long-term single use is commonly applied for the treatment of patients with all stages of estrogen-dependent breast cancer, including the premenopausal period [4]. The Japanese Breast Cancer Society currently recommends the single use of TAM for more than 5 years for premenopausal patients with estrogen-dependent breast cancer [5]).

On the other hand, the direct effects of TAM on ovarian function were proposed about half a century ago [6]. TAM was also reported to be used for controlled ovulation induction [7]. Based on these findings, TAM treatment during the premenopausal period was reported to be associated with ovarian stimulation, showing an increase of serum levels of estradiol and progesterone [8]. Persistent follicular functional cysts were also observed in premenopausal women with TAM treatment [9–12]. In addition, 2 premenopausal patients with ovarian hyperstimulation (OHS) showing multiple follicular formation were reported during TAM treatment, being accompanied by very high concentrations of serum estradiol of 1200 and 698.8 pg/mL [13].

Recently, we retrospectively analyzed 62 patients who received post-operative TAM therapy for endocrine-dependent breast cancer, and found that 11 patients showed high values of serum estradiol together with single or multiple follicular development [14]. From these findings, we proposed that dual mechanisms through the inhibition of both positive and negative feedback to the hypothalamic-pituitary-axis by TAM are responsible for OHS that represent single (by the inhibition of positive feedback) or multiple (by the inhibition of negative feedback) follicular development [14].

Since the mean concentration of serum estradiol (1015.8 pg/mL) in patients with OHS was very high and the incidence rate (17.7%) was not negligible [14], we further performed a retrospective multicenter study to analyze patients with breast cancer, who received post-operative TAM therapy combined with or without an anti-cancer drug or a Gn-RH agonist in order to obtain more precise information about risk factors for TAM-induced OHS.

## Methods

**Fig. 1:**
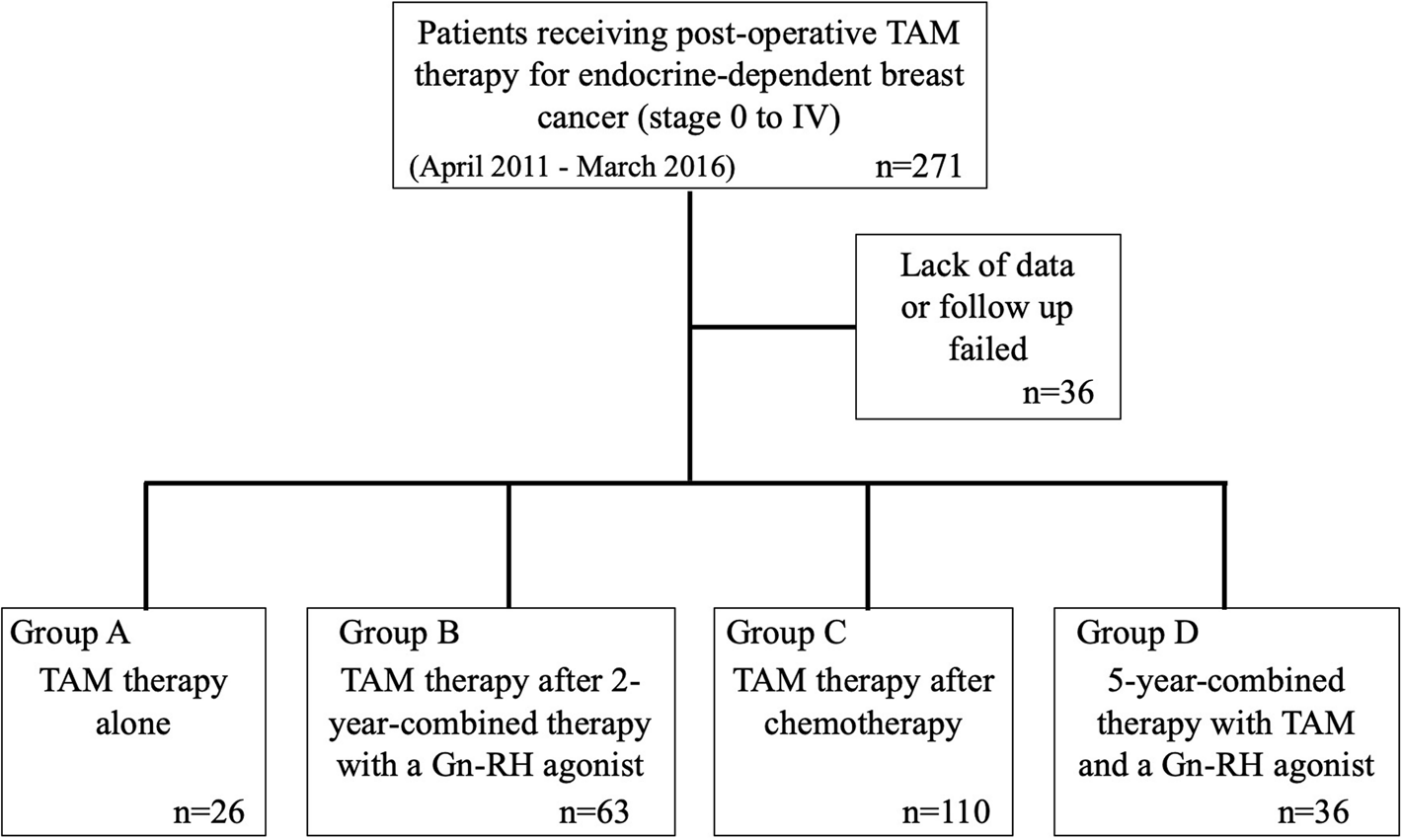
Flow chart showing the study design and groups. Two hundred and seventy-one premenopausal patients, who received post-operative TAM therapy for endocrine-dependent breast cancer (stage 0 to IV) from April 2011 to March 2016, were recruited in this study. Among them, 36 cases were omitted by lack of data or follow up failed. The remaining patients (*n* = 235) were classified into 4 groups based on pre- or post-operative adjuvant therapies: group A (*n* = 26), treated with TAM alone; group B (*n* = 63), TAM treatment after 2-year-combined therapy with a Gn-RH agonist; group C (*n* = 110), TAM treatment after chemotherapy; group D (*n* = 36), 5-year-combined therapy with TAM and a Gn-RH agonist

**Fig. 2:**
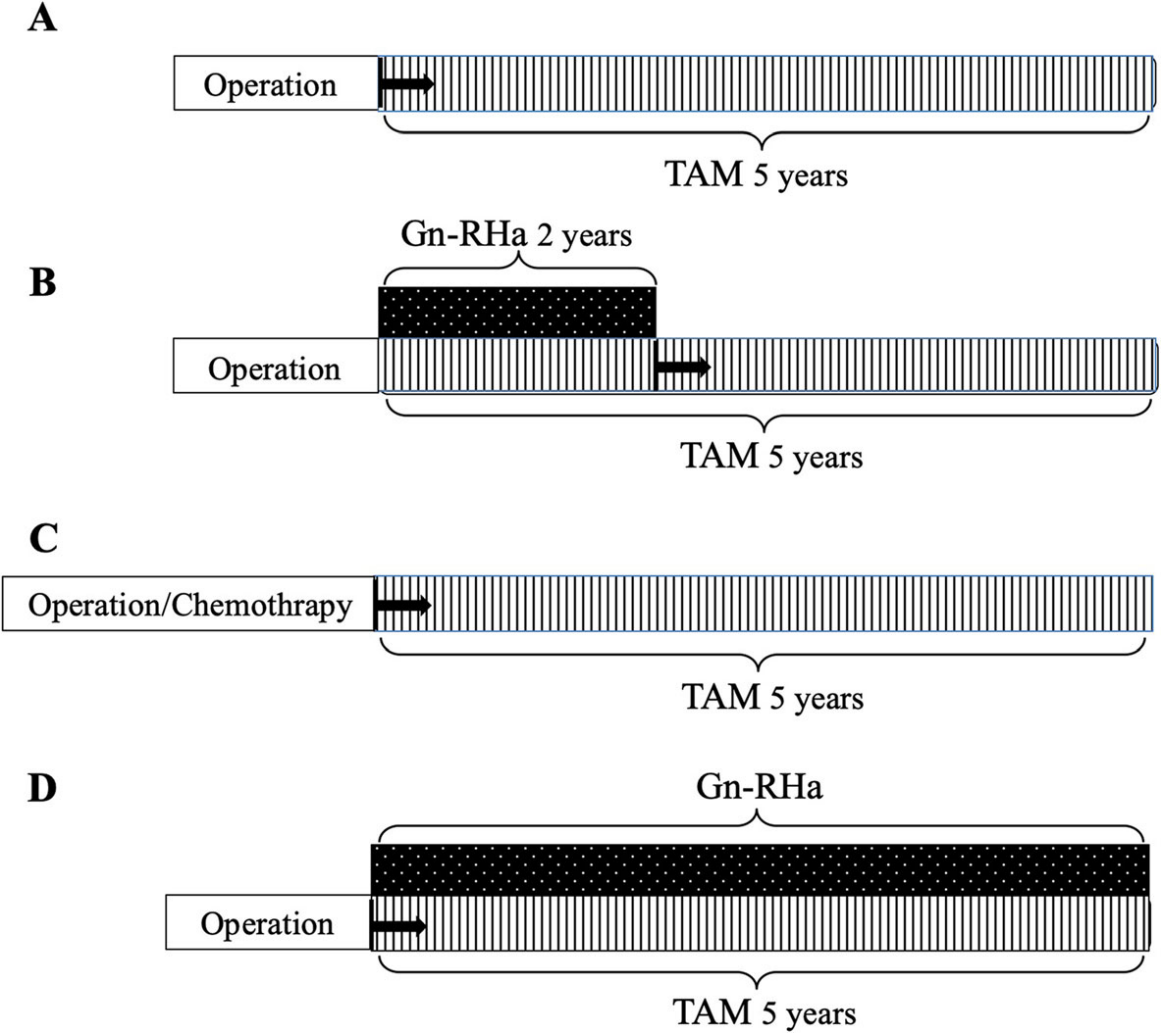
Therapeutic schedules with or without adjuvant therapy such as chemotherapy and Gn-RH analog treatment. The recruited patients were classified into 4 groups based on pre- or post-operative adjuvant therapies: group **a** (*n* = 26), treated with TAM alone; group **b** (*n* = 63), TAM treatment after 2-year- combined therapy with a Gn-RH agonist; group **c** (*n* = 110), TAM treatment after chemotherapy; group **d** (*n* = 36), 5-year-combined therapy with TAM and a Gn-RH agonist. In group **b**, clinical evaluation was started just after the end of 2-year-Gn-RH analog treatment, while the evaluation in group **d** was initiated from the start of Gn-RH analog treatment (black arrows). Black arrows, the start of evaluation

Two hundred and seventy-one premenopausal patients, who received periodic (6 months) measurement of serum concentrations of FSH and estradiol during post-operative TAM therapy for hormone receptor-positive endocrine-dependent breast cancer (stage 0 to IV) from April 2011 to March 2016 in the Department of Breast Oncology or Surgery of Kanazawa University, Tokushima University Graduate School, Teikyo University School of Medicine, or Kansai Medical University, were recruited in this study. Among them, 36 cases were omitted by lack of data or follow up failed (Fig. 1). The remaining patients (*n* = 235) were classified into 4 groups based on pre- or post-operative adjuvant therapies: group A (*n* = 26), treated with TAM alone; group B (*n* = 63), TAM treatment after 2-year-combined therapy with a Gn-RH agonist; group C (*n* = 110), TAM treatment after chemotherapy; group D (*n* = 36), 5-year-combined therapy with TAM and a Gn-RH agonist (Fig. 2). In group B, clinical evaluation was started just after the end of 2-year-Gn-RH analog treatment, while the evaluation in group D was initiated from the start of Gn-RH analog treatment (black arrows, Fig. 2b and d). The patients also consulted the corresponding Department of Gynecology based on a routine schedule to evaluate follicular development and endometrial hyperplasia by vaginal ultrasonographic examination.

In this study, a serum estradiol value of more than 300 pg/mL, which exceeds the normal estradiol production, or mean follicular diameter of more than 30 mm was defined as OHS. Estradiol and follicle-stimulating hormone (FSH) were measured by electro-chemiluminescence immunoassay (ECLIA) kits (Roche Diagnostics K.K., Tokyo, Japan). The differences in ages, estradiol, FSH, follicular diameters, and follicular numbers among the groups A-D were analyzed by the Kruskal-Wallis test. The differences in ages between OHS-positive and -negative groups were calculated by the Mann-Whitney *U*-test. The differences in the positive rate of OHS, the ratio of OHS following amenorrhea among the groups, were analyzed by the chi-square test and Ryan procedure as a post-hoc test [15]. The relationship between the incidence of OHS and age was analyzed by the chi-square test. The data of ages are shown as the median and interquartile range.

This retrospective study was approved by the Medical Ethics Committee of Kanazawa University (no. 1798), Tokushima University Graduate School (no. 2958–1), Teikyo University School of Medicine (no. 15–189), and Kansai Medical University (no. 2017003). General permission of use of clinical data was obtained from each patient before operation. Informed consent of using these clinical data for the specific aim of this study was additionally confirmed by the method of opt-out on the website from the patients, which was also approved by ethical committees.

## Results

### Differences in positive rates of OHS among the groups

**Fig. 3:**
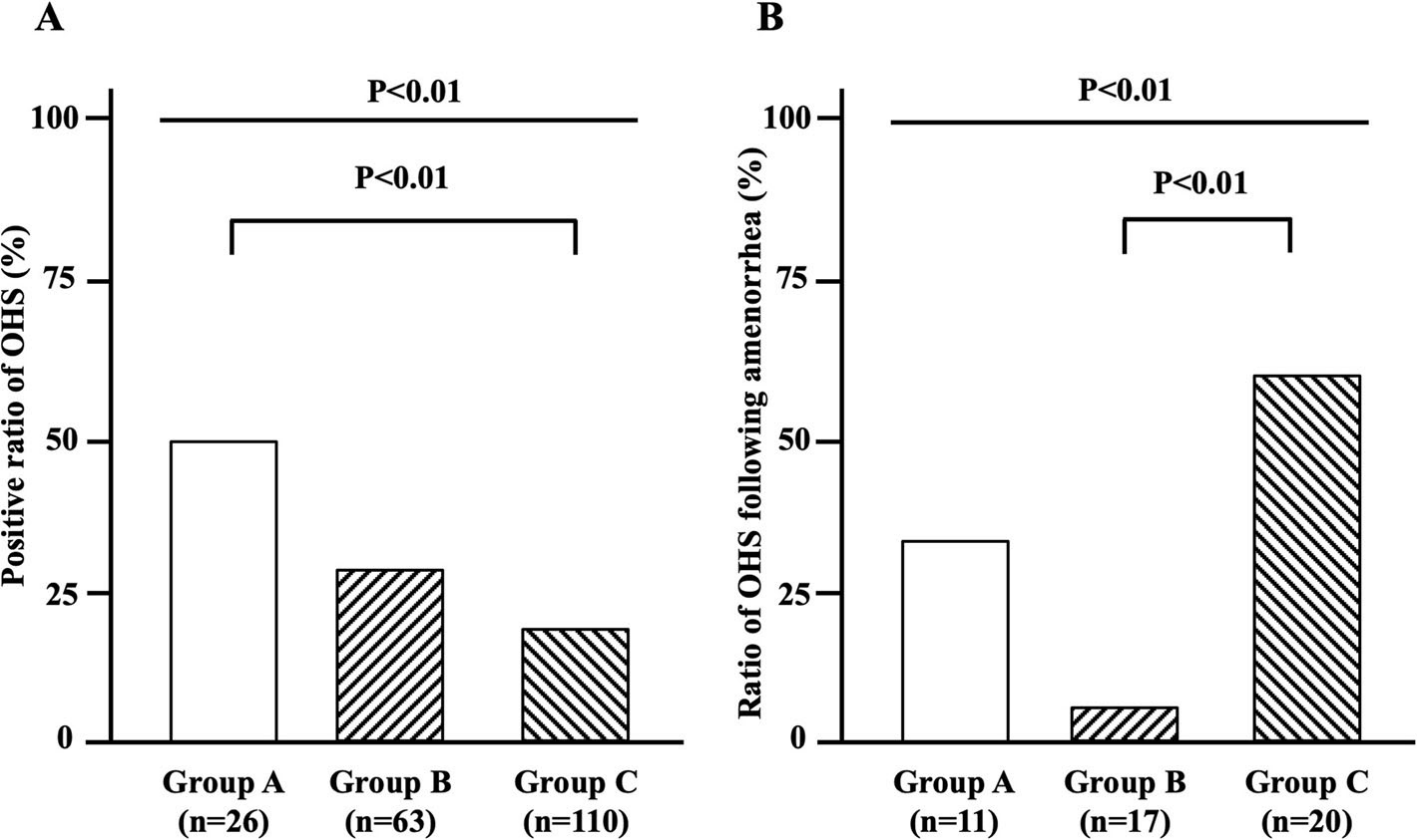
Differences in incidence rates of OHS among groups A-C. **a**, among groups A-C, in which TAM alone was administered to the patients during the analyzed periods, the overall positive ratio of OHS was 25.1% and its ratio in group A was significantly higher than that in group C. **b**, the rate of the onset of OHS following amenorrhea was significantly different among the three groups

The clinical profiles of 4 groups were shown in Table 1. The incidence of OHS was 50.0% in group A, 27.0% in group B, 18.2% in group C, and 0% in group D. Among groups A-C, in which TAM alone was administered to the patients during the analyzed periods, the overall positive rates of OHS was 25.1% and its rate in group A was significantly higher than that in group C (Fig. 3a). Notably, the rate of the onset of OHS following amenorrhea was significantly different among group A (*n* = 4/11, 36.6%), group B (*n* = 1/17, 5.9%), and group C (*n* = 13/20, 65.0%) (Fig. 3b). The history of an amenorrheic condition was unclear in 2 cases of OHS in group A, and they were omitted from Fig. 3b. In groups C and D, the regimens of chemotherapy were listed in Table 1. In group C, there was no significant difference in the positive rates for OHS among DOC+FEC (22.4%), TC (11.6%), AC-T (28.6%), and others (0%).

**Table 1:** Clinical profiles of patients included in this study

Patient background	Group A (*n* = 26)	Group B (*n* = 63)	Group C (*n* = 110)	Group D (*n* = 36)
Age (median and interquartile range)	45.5 (42–51)	46 (43–51)	45 (41–48)45	(39–47)
Stage (number)				
0	8	0	0	1
I	15	49	16	16
II	3	14	68	13
III	0	0	25	2
IV	0	0	1	1
Unclear	0	0	0	3
Chemotherapy (number)				
	0	0	110	18
DOC-FEC	–	–	58	4
DOC-EC	–	–	0	3
TC	–	–	43	0
EC	–	–	0	8
AC-T	–	–	7	1
AC	–	–	1	0
T	–	–	1	2

*DOC+FEC* (docetaxel followed by fluorouracil, epirubicin, cyclophosphamide), *EC* (epirubicin and cyclophosphamide), *TC* (docetaxel and cyclophosphamide), *AC-T* (adriamycin and cyclophosphamide-paclitaxel), *AC* (adriamycin and cyclophosphamide), *T* (paclitaxel)

### Relationship between the age and incidence of OHS

**Fig. 4:**
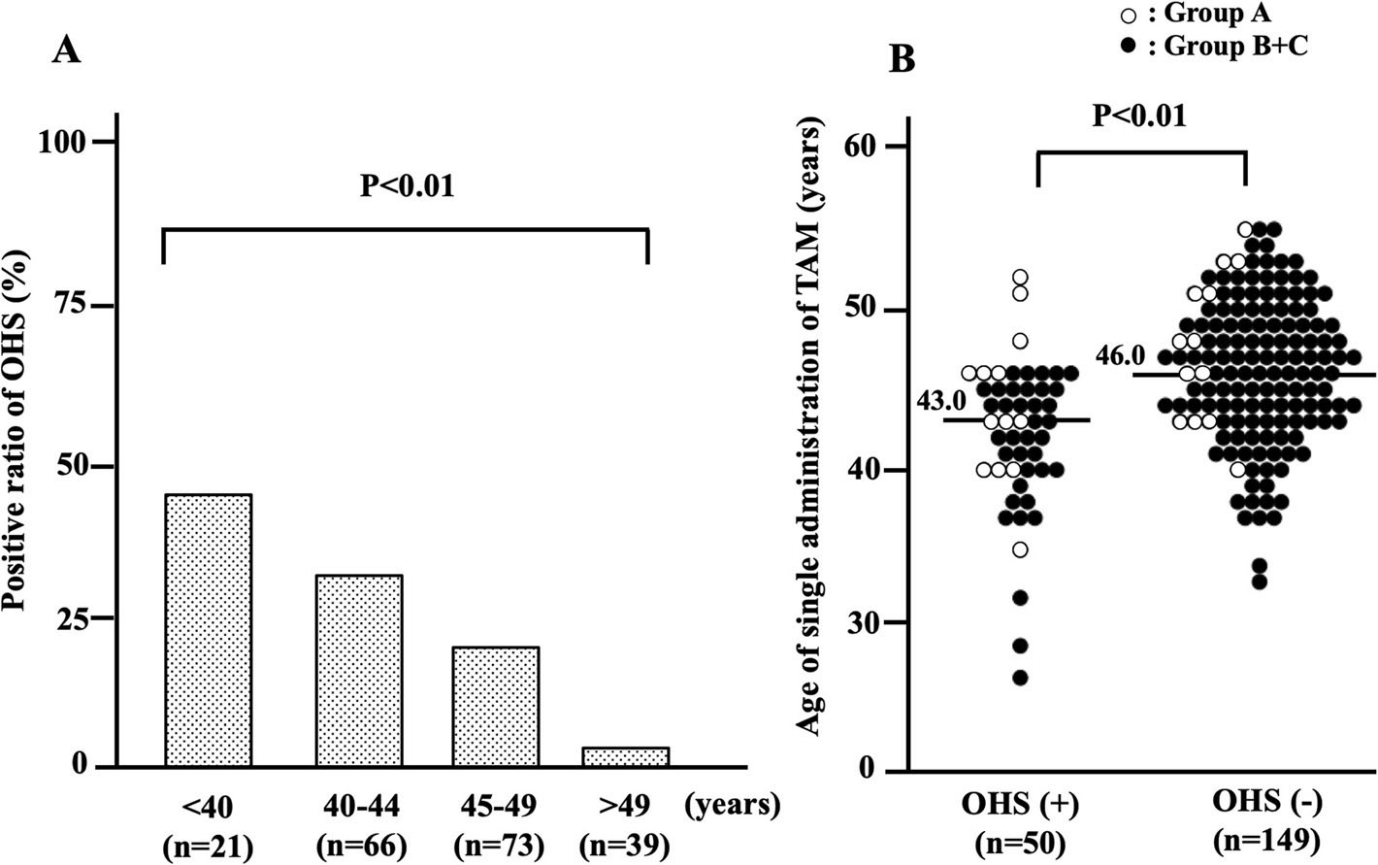
Relationship between ages and incidence of OHS. **a**, the incidence of OHS was significantly decreased according to aging. **b**, the median age of patients with OHS was significantly lower than that without OHS. Excluding 3 cases in group A, OHS-positive patients comprised a younger population of less than 47 years old

The median ages at the start of evaluation (Fig. 1, black arrows) in each group were 46 (43–51) in group A, 46 (43–50) in group B, 45 (41–48) in group C, and 45 (39–47) in group D. There were no significant differences among the groups. The incidence of OHS was significantly decreased according to aging from 47.6% (less than 40 years old) to 5.1% (no less than 50 years old) (Fig. 4a). The median age of patients with OHS was significantly lower than that of those without OHS. Based on these results, we estimated the valuable parameters for clinical use and paid an attention to the age at 47 years old because no OHS was observed in patients of no less than 47 years old in groups B and C. Only 3 cases in group A (3/50, 6%), which were over 47 years old, were positive for OHS (Fig. 4b). When the ages were limited to less than 47 years old, the incidence of OHS was 62.5% in group A (*n* = 10/16), 48.6% in group B (*n* = 17/35), and 28.2% in group C (*n* = 20/71).

### Differences in hormonal concentration, follicular diameters, and numbers of OHS patients among the groups

**Fig. 5:**
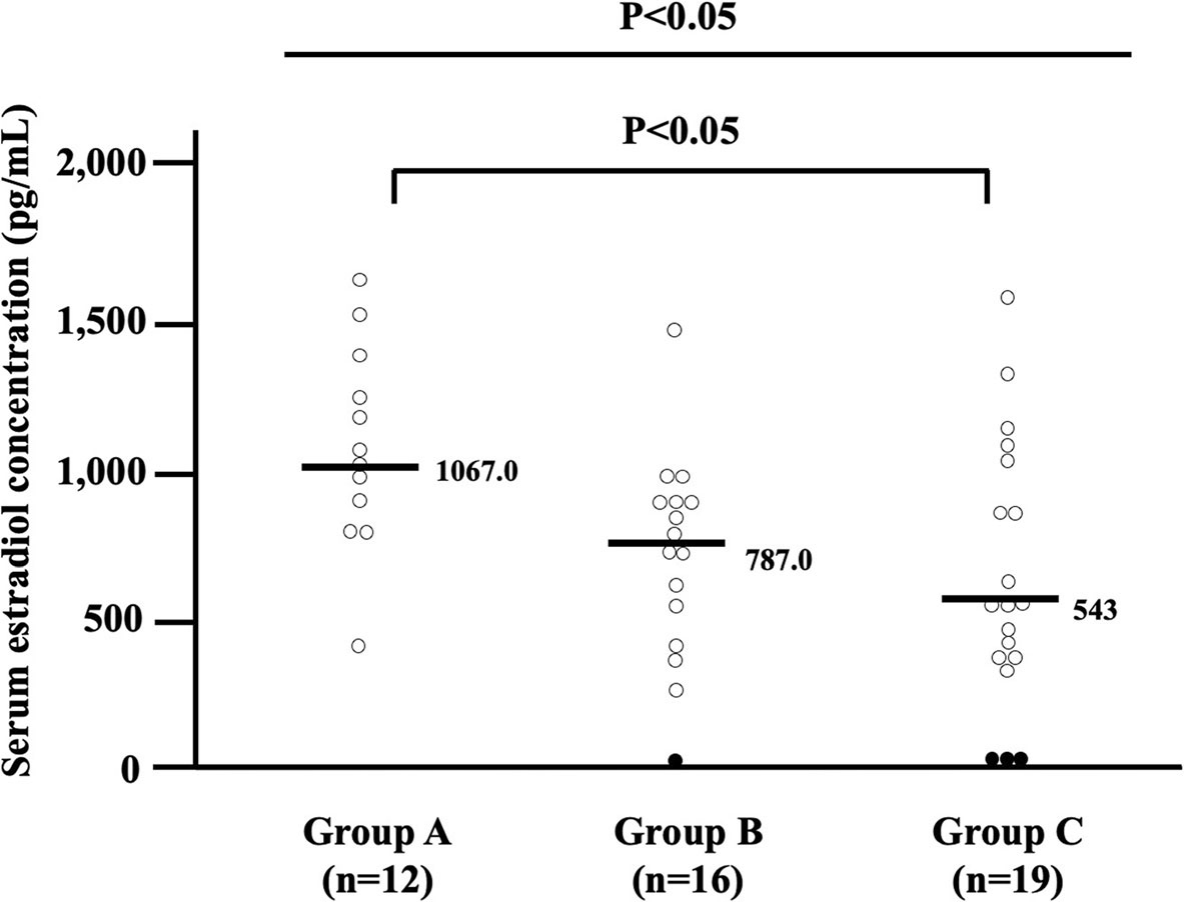
Differences in estradiol concentration among groups **a-c**. The median serum concentrations of estradiol in OHS were 1067 pg/mL in group A, 787.0 pg/mL in group B, and 543.0 pg/mL in group C. In 4 of 26 patients with ovarian cyst formation, the serum concentration of estradiol was very low

The median serum concentrations of estradiol in the presence of OHS were 1067 pg/mL in group A, 787.0 pg/mL in group B, and 543.0 pg/mL in group C (Fig. 5). In each group, one case missed the value of the estradiol concentration, and these 3 cases were omitted from Fig. 5. In 4 of 26 cases with ovarian cyst formation, the serum concentration of estradiol was very low, which may represent a past history of OHS (filled circles in Fig. 5). There was no significant difference in FSH concentrations (8.25 mIU/mL, 8.25 mIU/mL, and 11.2 mIU/mL, respectively), follicular diameters (34.5 mm, 50.5 mm, and 43.0 mm, respectively) or follicular numbers (1.33, 1.18, and 1.38, respectively) at the time of detection of OHS among groups A-C.

## Discussion

**Fig. 6:**
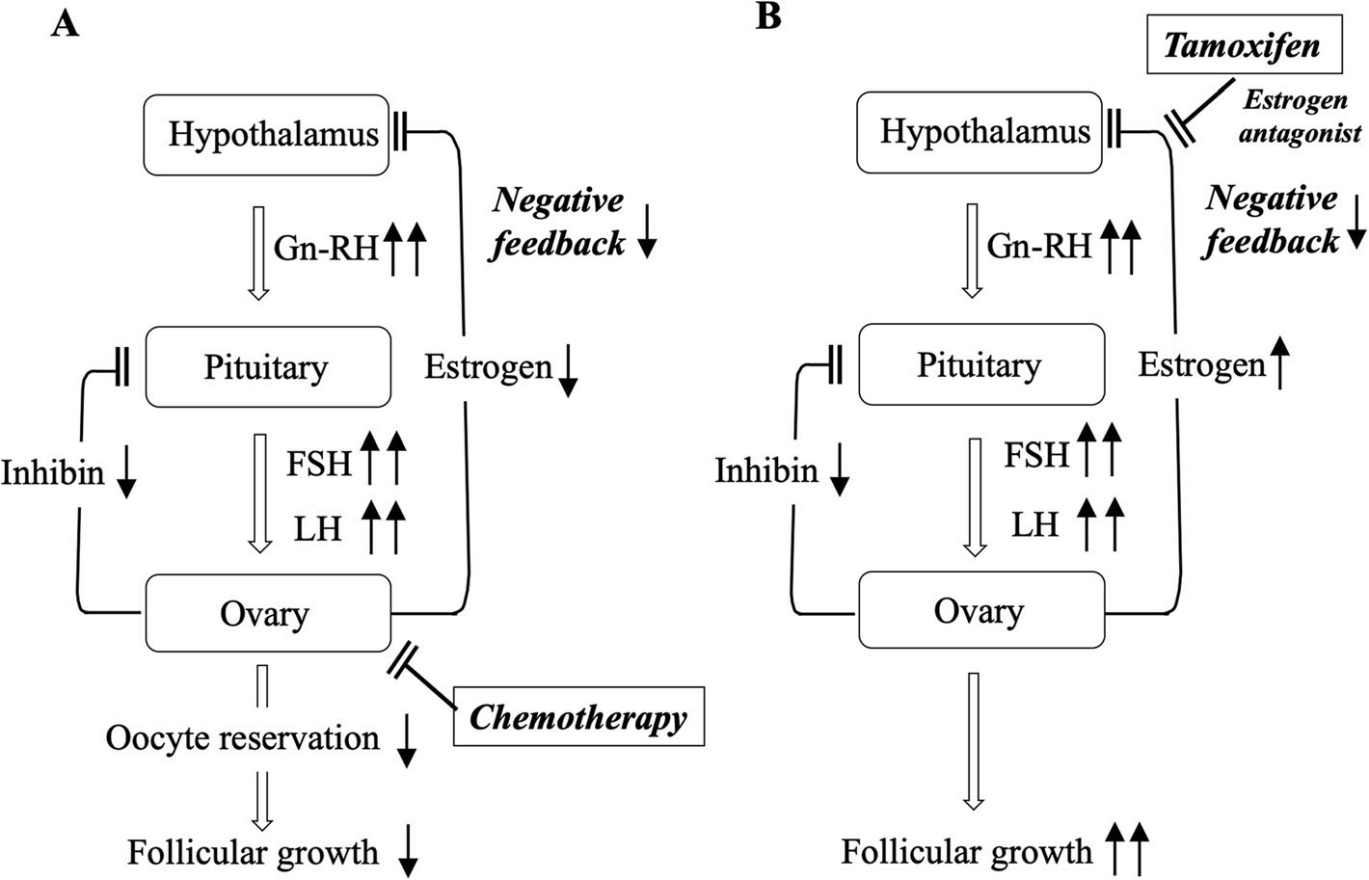
Hypothalamic-pituitary-ovarian axis in group C. **a**, chemotherapy can damage the ovarian oocyte reserve and subsequently reduce inhibin production from ovarian follicles, decreasing the direct suppression of FSH secretion from the pituitary. By a negative feedback mechanism through the hypothalamic-pituitary axis, low production of estradiol and administration of anti-estrogenic TAM can induce high-level secretion of gonadotropins, concomitant with loss of the direct suppressive effects of inhibin on FSH secretion. **b**, at the regrowth of follicles after chemotherapy, since negative feedback is inhibited by TAM, the re-activated process of follicular growth can be accelerated by the excessive secretion of gonadotropins under these conditions, explaining the high incidence of OHS following amenorrhea

In this study, we confirmed the stimulatory effects of TAM on the ovarian function during treatment for breast cancer in Japanese women of reproductive age. To our knowledge, this study is the first to focus on the analysis of differences in the incidence of OHS among TAM-based adjuvant therapies for breast cancer in premenopausal women. The positive rates of OHS induced by TAM were markedly different among the therapeutic schedules with or without combined therapy. As expected, the risk of OHS in group D was very low, indicating that the parallel use of a Gn-RH analog can overcome the adverse effects of TAM on ovarian functions. This result also supports the theoretical mechanism whereby its effects on the ovarian function are induced through the hypothalamic-pituitary axis as an estrogen antagonist (Fig. 6b) [13, 14].

Although recent reports also pointed out the stimulatory effects TAM on the ovarian function [16, 17], its precise incidence rate remains unclear. The positive rate of OHS among groups A-C (25.1%) was slightly higher than the ratio estimated in our previous report [14]. Since this rate in group A reached 50%, the individual risk of OHS should be considered when following-up patients with breast cancer during post-operative TAM therapy. In this study, as the routine follow-up was performed biannually, the real incidence rates may be higher. It should also be noted that the median serum concentration of estradiol in OHS was very high, which is probably too excessive for TAM to block estrogenic effects. Importantly, the incidence of OHS is significantly correlated with aging. Among patients no less than 47 years old, since it was absent in groups B and C (*n* = 0/67, 0%), the risk of OHS may be very low in these groups. However, when the age was limited to less than 40 years, the positive rate of OHS was 45% in groups B and C, suggesting the need for careful follow-up even in these groups. Notably, although the number is small, the positive rate of OHS was relatively high in group A (28.6%) at more than 50 years old. Since ovarian aging is influenced by various clinical histories such as previous ovarian surgeries, polycystic ovarian syndrome, endometriosis, and infertility, it may be valuable in the next step to investigate the effects of these histories, for example, on the difference in recovery period from chemotherapy between patients with and without endometriosis.

In a previous study, in 4 patients diagnosed with chemotherapy-induced menopause, a high value of serum estradiol was observed during the amenorrheic period that had continued from chemotherapy, suggesting that anovulatory hyperestrogenic conditions may be induced by TAM treatment [14]. In accordance with our speculation, this study clearly demonstrated that the ratio of the onset of OHS following amenorrhea was very high in group C (65.0%), suggesting that we should meticulously observe patients in the post-chemotherapeutic period during TAM treatment. Since chemotherapy can damage the ovarian oocyte reserve [18], it subsequently reduces inhibin production from ovarian follicles, decreasing the direct suppression of FSH secretion from the pituitary [19, 20] (Fig. 6a). By a negative feedback mechanism through the hypothalamic-pituitary axis, low production of estradiol and administration of anti-estrogenic TAM can induce high-level secretion of gonadotropins, concomitant with the loss of the direct suppressive effects of inhibin on FSH secretion (Fig. 6a). At the regrowth of follicles after chemotherapy, since negative feedback is inhibited by tamoxifen, we speculate that the re-activated process of follicular growth can be accelerated by the excessive secretion of gonadotropins under these conditions, explaining the high incidence of OHS following amenorrhea after chemotherapy (Fig. 6b). Accordingly, we should note that estrogenic conditions can dramatically change during the amenorrheic period especially in group C.

**Fig. 7:**
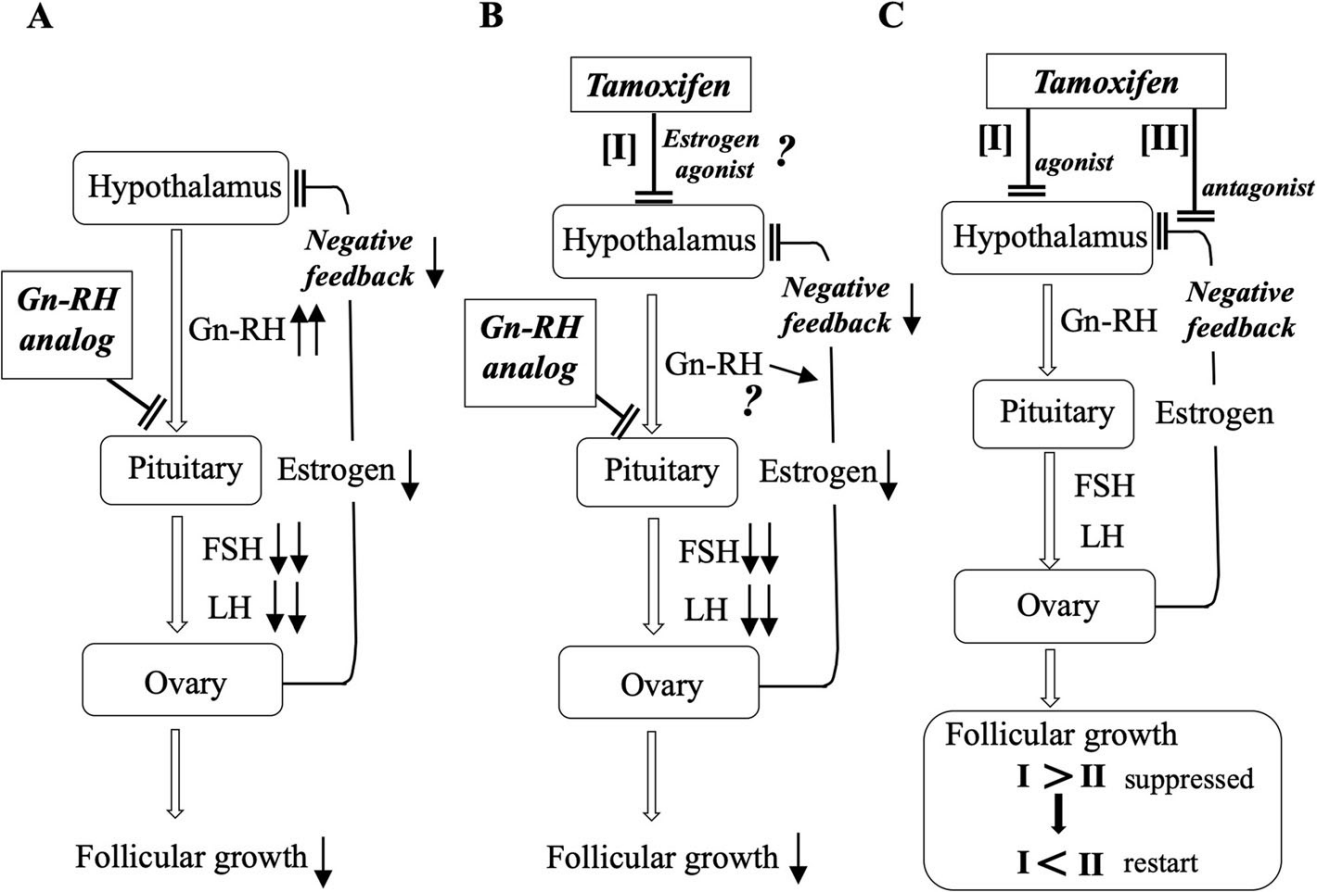
Hypothalamic-pituitary-ovarian axis in group B. **a**, mechanisms of ovarian suppression by the Gn-RH analog in women of reproductive age. The Gn-RH analog causes the down-regulation of Gn-RH receptors in pituitary cells, which then induces a decrease of gonadotropin secretion and the subsequent low production of estrogen in the ovary. Under these low-estrogen conditions, Gn-RH secretion in the hypothalamus may be accelerated. **b** and **c**, possible explanation for the delayed resumption of menses in women during TAM administration after combined Gn-RH analog treatment. **b**, the delayed resumption of menses during TAM administration after combined Gn-RH analog treatment suggests transient suppressing effects of TAM on Gn-RH secretion in the hypothalamus (see pathway [I]). TAM acts on the hypothalamus as an estrogenic modulator under the low-estrogen conditions during Gn-RH analog treatment, suppressing high secretion of Gn-RH in the hypothalamus. **c**, the suppressing effects of TAM persist for over half a year even after cessation of the Gn-RH analog. When the balance between the hypothalamus-suppressing effect [I] and negative feedback-inhibitory effect (see pathway [II]) become converted, normal follicular growth and menses gradually restart and then OHS will occur

In contrast to group C, the rate of the onset of OHS following amenorrhea was very low in group B (Fig. 5). In general, treatment using a Gn-RH analog alone for women of reproductive age is considered to cause the down-regulation of Gn-RH receptors in pituitary cells, which then induces a decrease of gonadotropin secretion and a subsequent low production of estrogen in the ovary. Under these low-estrogen conditions, Gn-RH secretion in the hypothalamus is speculated to be accelerated (Fig. 7a). After cessation of the Gn-RH analog, the expression of Gn-RH receptors will recover and gonadotropin secretion and follicular development will be gradually promoted, achieving the resumption of menses at least within 6 months [21]. In contrast to treatment by Gn-RH analog alone, the resumption of menses was not observed within 6 months in almost all cases in group B after terminating Gn-RH analog administration. Although the precise mechanisms remain unknown, the delayed resumption of menses during TAM administration after combined Gn-RH analog treatment suggests transient suppressing effects of TAM on Gn-RH secretion in the hypothalamus. To explain this intriguing finding, we suppose that TAM acts on the hypothalamus as an estrogenic agonist under the low-estrogen conditions during Gn-RH analog treatment, suppressing high secretion of Gn-RH in the hypothalamus (Fig. 7b), and continuing this effect over half a year even after cessation of the Gn-RH analog (Fig. 7c). When the balance between hypothalamus-suppressing (Fig. 7c, see pathway [I]) and negative feedback-inhibitory effects (Fig. 7c, [II]) become converted, normal follicular growth and menses gradually restart and then OHS will occur, characterizing the low ratio of the onset of OHS following amenorrhea in group B.

## Conclusion

This study confirmed that TAM can induce OHS during treatment for breast cancer in Japanese women of reproductive age. The median serum concentration of estradiol in the presence of OHS was very high, which TAM cannot completely block as an anti-estrogenic agent. The incidence rate of OHS was inversely correlated with the age and markedly different among the therapeutic schedules with or without adjuvant therapy such as chemotherapy and Gn-RH analog treatment. When being treated with TAM alone (group A), the incidence rate of OHS reached 50%. The rate of the onset of OHS following amenorrhea was very high after chemotherapy, while its rate was very low after Gn-RH analog treatment, suggesting that combined treatment-based management of TAM therapy should be recommended. Consequently, we should be cautious regarding the onset of OHS in young women, especially in patients during the amenorrheic period after chemotherapy.

The relationship of the incidence of high estrogenic condition and risk for relapse of breast cancer should be investigated in the future. Additionally, it was recently reported that breast cancer survivors treated with tamoxifen showed low rate of having a child compared with the tamoxifen nonusers [22]. This study also demonstrated that ovarian reserve rates in the tamoxifen users were higher than those in the tamoxifen nonusers, suggesting the presence of non-ovarian factors to induce the adverse effects on fertility by TAM treatment. Considering the direct cancer-inducing effects of TAM on endometrium [23], we should note a novel possibility that TAM directly impairs endometrial functions to receive embryo implantation under the hyperestrogenic conditions, especially in the younger patients who desire to conceive after breast cancer therapy. Consequently, further accumulation of clinical information on these issues must be obtained through co-operation between breast surgeons and gynecologists.

## Data Availability

All data generated or analyzed during this study are included in this published article.

## Abbreviations

FSH: Follicle-stimulating hormone
Gn-RH: Gonadotropin-releasing hormone
LH: Luteinizing hormone
OHS: Ovarian hyperstimulation
TAM: Tamoxifen
